# Point-of-Care Diagnostic Tools for Surveillance of SARS-CoV-2 Infections

**DOI:** 10.3389/fpubh.2021.766871

**Published:** 2021-11-25

**Authors:** Dhanasekaran Sakthivel, David Delgado-Diaz, Laura McArthur, William Hopper, Jack S. Richards, Charles A. Narh

**Affiliations:** ^1^ZiP Diagnostics Pty Ltd., Collingwood, VIC, Australia; ^2^School of Medicine, Monash University, Clayton, VIC, Australia; ^3^Department of Life Sciences, Burnet Institute for Medical Research, Melbourne, VIC, Australia; ^4^Department of Medicine, University of Melbourne, Melbourne, VIC, Australia

**Keywords:** COVID-19, SARS-CoV-2, point-of-care, diagnostics, isothermal amplification (LAMP), sample types, surveillance

## Abstract

Severe acute respiratory syndrome coronavirus-2 (SARS-CoV-2) is a recently emerged and highly contagious virus that causes coronavirus disease 2019 (COVID-19). As of August 24, 2021, there were more than 212 million confirmed COVID-19 cases and nearly 4.4 million deaths reported globally. Early diagnosis and isolation of infected individuals remains one of the most effective public health interventions to control SARS-CoV-2 spread and for effective clinical management of COVID-19 cases. Currently, SARS-CoV-2 infection is diagnosed presumptively based on clinical symptoms and confirmed by detecting the viral RNA in respiratory samples using reverse transcription polymerase chain reaction (RT-PCR). Standard RT-PCR protocols are time consuming, expensive, and technically demanding, which makes them a poor choice for large scale and point-of-care screening in resource-poor settings. Recently developed isothermal nucleic acid amplification tests (iNAAT), antigen and/or serological tests are cost-effective to scale COVID-19 testing at the point-of-care (PoC) and for surveillance activities. This review discusses the development of rapid PoC molecular tools for the detection and surveillance of SARS-CoV-2 infections.

## Introduction

The diagnosis of active SARS-CoV-2 infection is critical in epidemiological surveillance, infection control and contact tracing, clinical management, and for monitoring the impact of interventions against the spread of the virus. Current diagnostic tests fall into three main categories: molecular tests that detect the SARS-CoV-2 RNA, antigen tests that detect the presence of specific viral antigens, and serological tests that detect anti-SARS-CoV-2 immunoglobulins (Ig). COVID-19 diagnosis criteria vary among countries (1) but in every case, detection of SARS-CoV-2 RNA by reverse transcriptase PCR (RT-PCR) is considered a confirmatory diagnosis (2). However, RT-PCR is expensive and laborious; requiring viral RNA isolation, purification and reverse transcription to complementary DNA (cDNA) before amplification using PCR. Hence, it requires skilled personnel and dedicated laboratory space, thus limiting its use in resource-limited settings. More recently, serology and antigen-based test, and isothermal nucleic acid amplification test (iNAAT) have become available for the diagnosis of COVID-19 (Table 1). These tests have acceptable sensitivity and do not require sophisticated equipment, offer rapid turnaround time within an hour and can be performed at the point-of-care (PoC). To date, several RT-PCR diagnostic kits and, to a lesser extent antigen/antibody-based detection tests, isothermal amplification tests, clustered regularly interspaced short palindromic repeats (CRISPR-) and sequencing-based detection tools have been approved by the Food and Drug Administration (FDA) via Emergency Use Authorization (EUA) for the diagnosis of COVID-19. This review discusses the molecular, serological, and antigen diagnostic tools for detecting SARS-CoV-2 infections, their potential use for PoC diagnosis of COVID-19 (Table 1).

**Table 1:** Attributes and performance of NAAT and antigen tests used for COVID-19 diagnosis.

		**RT-LAMP**	**RT-PCR (Gold standard)**	**Antigen assay**	**References**
Sample types used	Upper respiratory: saliva, nasopharyngeal/ oropharyngeal swabs	Yes	Yes	Yes	(95–97)
	Lower respiratory: sputum, tracheal/bronchoalveolar aspirates	Yes	Yes	Yes	(94, 98–100)
Sample preparation/input	Crude sample preparation with lysate as input for amplification	Yes (cell lysates in lysis buffer compatible with LAMP)	Yes, but not used routinely in diagnostic labs as it may reduce assay sensitivity	Yes. cell lysates in suitable lysis buffer compatible with antigen assay	(96, 101–105)
	Purified RNA as input for amplification	Yes, RNA is extracted and purified using in-house reagents or commercial kits	Yes, RNA is routinely purified, particularly for clinical diagnosis	Antigen detection assay detects the SARS-CoV-2 surface proteins in the lysate	(37, 106, 107)
Test technology	cDNA synthesis and amplification in the same reaction.	Yes; with commercially available reverse transcriptase and Bst DNA polymerase (possess strand displacement activity). E.g., NEB RT-LAMP mix	Yes; with commercially available reverse transcriptase and Taq DNA polymerase. E.g., TaqMan^TM^ SARS-CoV-2 RT-PCR assay kit	N/A	(65, 87, 94, 97, 102, 108–110)
	Number of primers used	At least 4 primers. Optional inclusion of 2 loop primers to speed up amplification	At least 2 primers. Optional inclusion of probe for real-time amplicon detection	Antigen detection assay uses monoclonal and/or polyclonal antibody specific to SARS-CoV-2 antigen	
	Detection of multiple gene targets	Usually, 2–3 gene targets can be multiplexed in a single reaction tube. E.g., N and E gene	More than 2 gene targets can be multiplexed using fluorescent labeled primers or probes. E.g., E and RdRP gene	Targets viral proteins including spike and nucleocapsid.	
	RNA extraction, cDNA synthesis and amplification in a single reaction tube.	Yes, LAMP compatible lysis buffers can be used to lyse the virus in respiratory samples	Possible but not routinely used in diagnosis due to potential impact on assay sensitivity	N/A	
Detection modality	Instrumentation	Isothermal instrument (e.g., water bath/heat block)	Conventional/real-time PCR	Visual display of test results. Optional RDT reader	(103, 111)
	Amplicon detection	Use of DNA intercalating dyes; color change and/or fluorescence detection, turbidity (magnesium pyrophosphate formation)	Fluorescence from DNA intercalating dyes or probes.	N/A	(87, 101, 108, 112)
	Real-time detection	Yes (colorimetry and fluorescence detection)	Yes, fluorescence detection	Colorimetry and fluorescence detection	(87, 94)
	Sample-to-result	≤ 1 h	≥2 h	≤ 0.5 h	(111)
Analytical performance	Sensitivity	>95%	>93%	–	(11)
	Specificity	>98%	>95%	–	
Clinical performance	Sensitivity	>94%	>90%	75.8–100%	
	Specificity	>97%	>95%	90–100%	
Technological access	Skill requirement and point-of-care deployability	Minimal training with basic laboratory requirements at the point-of-care; e.g., Clinics	Technical expertise in PCR and require well-equipped laboratory; Accredited research laboratories and hospitals	RDTs are user-friendly and test can be performed at home	(52, 91, 111, 113–116)

*RT-LAMP, reverse transcription loop-mediated isothermal amplification; RT-PCR, reverse transcription polymerase chain reaction; RNA, ribonucleic acid; cDNA, complementary deoxyribonucleic acid; NEB, New England BioLabs; N/A, not applicable. Analytical and Clinical validation data taken from the European Commission COVID-19 in vitro diagnostic devices and test methods database (111)*.

## Antibody- and Antigen-Based COVID-19 Testing

Our understanding of immune response against SARS-CoV-2 infections has rapidly unfolded as millions of individuals have been infected. Seroconversion in infected individuals has been observed between 1 and 2 weeks post-symptom onset (3–9). Studies on the immune responses of SARS-CoV-2-infected patients have shown increased presence of follicular helper T cells, activated CD4^+^ and CD8^+^ T cells with the detection of Immunoglobulin A (IgA), IgM, and IgG against the SARS-CoV-2 spike (S), nucleocapsid (N) and envelop (E) proteins (6, 10–13). Antibodies against SARS-CoV-2 have been shown to persist at least 12 months post-infection in most individuals (14–18). The majority of SARS-CoV-2 rapid diagnostic tests detect the presence of anti-SARS-CoV-2 antibodies (IgG, and/or IgM) in plasma or serum of infected individuals (FDA.gov).

Immunoassays for COVID-19 diagnosis target the most immunogenic proteins—N and S (6, 10, 11, 19–21). In serum and plasma specimens, anti-SARS-CoV-2 antibodies could be detected as early as 2 weeks post-symptom onset (21). However, infected individuals show different antibody profiles over the course of the disease (12, 22). SARS-CoV-2-specific IgA and IgM antibodies have been detected 5 days post-symptom onset while IgG was detected 14 days post-symptom onset (12, 22), indicating early and evolving infections, respectively. The majority of the immunoassays in use are based on enzyme linked immunosorbent assay (ELISA), immunochromatography (lateral-flow) and antigen microarray (6, 10, 20–23). ELISA offers high-throughput but requires experienced technicians, a laboratory space, and several other instruments, and thus, it is not feasible for PoC diagnosis. In contrast, lateral-flow-based assays are easy to use, do not require instruments, and have been developed and deployed as PoC tools for serological and antigen-based diagnosis of COVID-19 (24, 25).

In spite of their advantages (Table 1), serological tests are limited in the diagnosis of SARS-CoV-2 infections due to their poor sensitivity to detect mild and asymptomatic infections (26). In addition, reports of individuals who remain PCR-positive after seroconversion suggest that they may still be shedding viral RNA during the convalescent stage. However, this may not necessarily indicate the presence of viable virus (3, 4, 27–30). Therefore, serological tests may be limited to identification of past but not active infections. Considering their relatively lower cost and ease of use in comparison with RT-PCR, they could be used to initially screen vulnerable populations to estimate seropositivity rates.

In contrast to antibody tests, antigen tests detect the presence of specific SARS-CoV-2 antigens in respiratory samples. Oropharyngeal, nasal and nasopharyngeal specimens are the most compatible specimen types for the majority of COVID-19 antigen and NAAT methods (2, 31). Antigen tests are relatively more affordable than RT-PCR, and considerably sensitive when used during the infectious period of the disease (32, 33). They are recommended for routine testing among at risk populations (34, 35). To date, over 20 SARS-CoV-2 antigen tests have received FDA emergency use authorization, reporting analytical sensitivities down to 30 TCID50/mL and specificities of up to 99% (36).

## Molecular Detection of SARS-CoV-2 Infections

**Figure 1:**
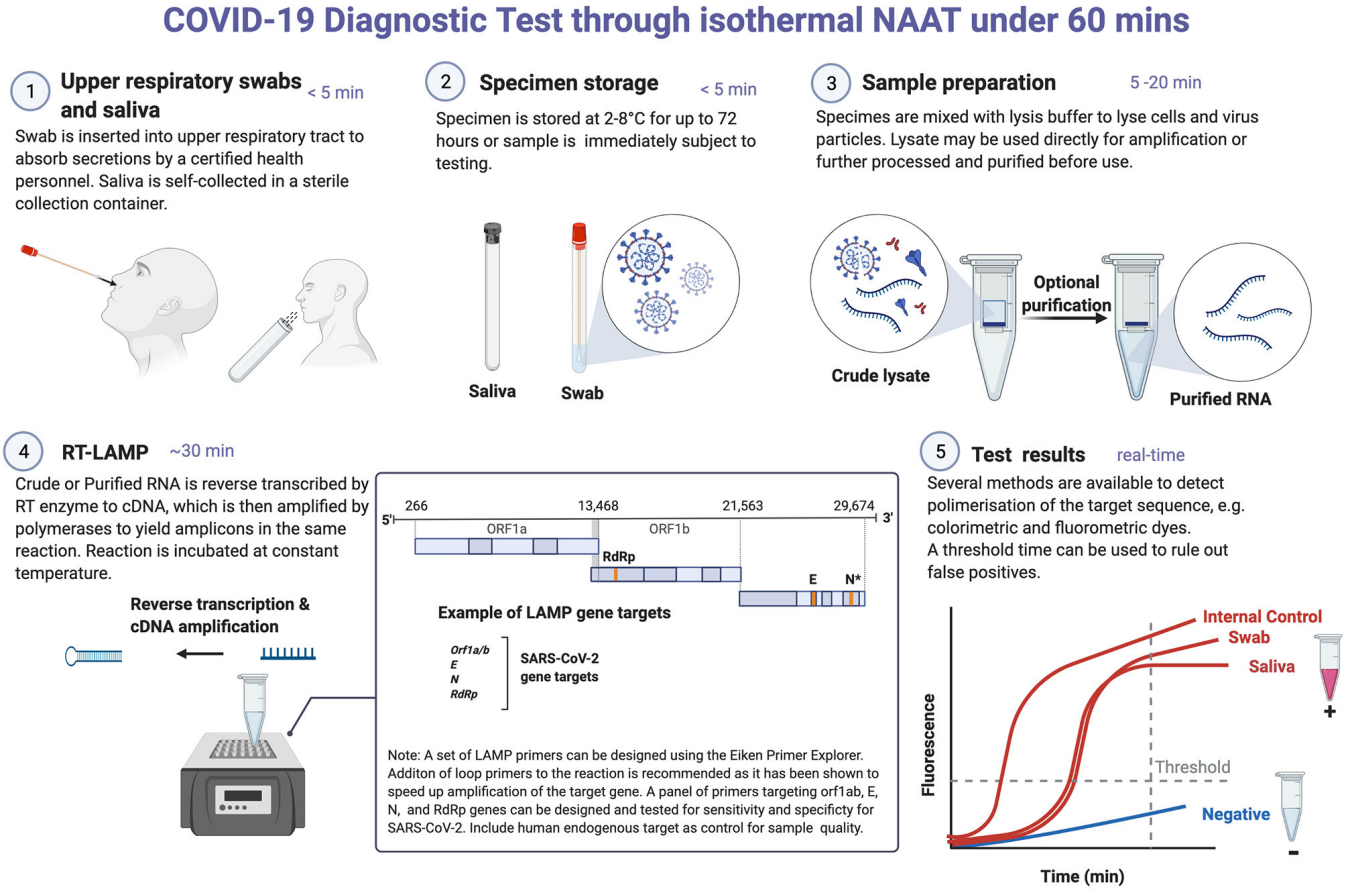
COVID-19 diagnostic testing through isothermal NAAT. Reverse transcriptase LAMP (RT-LAMP) detection of SARS-CoV-2 RNA in nasal swab and saliva samples. These samples can be stored in the refrigerator for 3 days prior to NAAT testing. Where testing can be done immediately, sample preparation and/or RNA extraction is performed, which may take between 5 and 20 min. RNA purification is often required for RT-PCR-based testing. This is done because contaminants in crude cell lysates could potentially reduce the polymerase activity of the reverse transcriptase and DNA polymerases used in RT-PCR. In contrast, the Bacillus stearothermophilus (Bst) DNA polymerase used in LAMP is more tolerant to inhibitors. For RT-LAMP testing, a set of four to six primers targeting any of the viral genes can be designed using online programs, e.g., Eiken Primer design software. It is recommended that a primer set targeting a human endogenous gene is included as a control for sample preparation/RNA extraction and amplification efficiency. In RT-LAMP, both cDNA synthesis (reverse transcriptase) and amplification (Bst) occur simultaneously and in the same reaction tube at a constant temperature (60–65°C). A double-stranded DNA intercalating dye can be added to detect amplicons either by colorimetry (show color change), fluorescent (for real-time detection) or both. RT-LAMP is prone to false-positive amplifications and as such any assay developed using this technique needs to be standardized for each test type. *In a NAAT-based assay, two targets on N gene were included in a single reaction to increase the test sensitivity. Created with Biorender.com.

Several NAAT tools have been developed to detect SARS-CoV-2 infections by amplifying the viral RNA from a wide range of sample types including nasal swabs and saliva samples. While RT-PCR is currently the gold standard for the detection of SARS-CoV-2 RNA due to its high sensitivity, other methods including recombinase polymerase amplification (RPA) and loop mediated isothermal amplification (LAMP, see Figure 1) have also been used for COVID-19 diagnosis (37–39). RT-PCR is expensive, laborious and requires skilled personnel, making it unsuitable for PoC diagnosis (37–39). The accuracy and sensitivity of RT-PCR is affected by the purity of the sample and/or extracted RNA. The global shortage of RNA extraction kits has had adverse impacts on COVID-19 diagnosis and control worldwide. Though extraction-free RT-qPCR protocols have been considered as alternatives for the standard SARS-CoV-2 RT-PCR method, laboratory optimizations are often required to minimize false-negative rates (40).

Several reports suggest that RT-PCR positivity does not necessarily correlate with clinical infectivity since respiratory samples may contain non-viable virus, which could persist in the body for several weeks during the convalescent stage of the disease (41–44). Viable virus can be cultured from samples with low RT-PCR cycle threshold (Ct) values while samples with high Ct values are less likely to contain culturable virus (3, 45–47), suggesting that individuals with high Ct values (usually those at later stages of the disease) are less likely to spread the virus than those with low Ct values (usually those in the acute phase of the disease).

The performance of NAATs on the detection of SARS-CoV-2 RNA has been extensively reviewed elsewhere (48–52). Due to the lack of standardization in the NAAT testing algorithms including sample types and target genes, it is difficult to compare the analytical performance of the various test types (Table 1). A study by Vogels and colleagues found that test sensitivity was comparable among most of the primer-probe sets with the exception of primer sets targeting the RNA dependent RNA polymerase (RdRp-SARSr) gene segment, which resulted in lower sensitivity (53). Significant difference in sensitivity has been observed with commercial RT-PCR kits. For instance, Igloi and colleagues evaluated 13 commercial kits and reported analytical sensitivities that ranged from 3.3 to 330 viral RNA copies in the RT-PCR assays evaluated (54).

Published gene targets for the detection of SARS-CoV-2 have comparable specificity. While RT-PCR based commercial kits used by the China National Institute for Viral Disease Control and Prevention (CCDC) predominantly target the Open Reading Frame 1ab (*orf1ab*) and N genes (55), other commercially available RT-PCR kits target the RNA-dependent RNA polymerase (*RdRP*) and/or E genes (37). In other assays, multiple targets on the same gene are included in order to increase the test sensitivity, e.g., two targets on the N gene (56). The sensitivity of PCR-based detection has been improved with double strand excision of the target using the CRISPR gene-editing technique (38, 39, 57). The rising number of mutations in the viral genome, particularly in the S and *orf1ab* genes have raised concerns about the sensitivity of NAAT tools to detect SARS-CoV-2 including emerged variants—alpha (B.1.1.7), beta (B1.3.51), gamma (P.1), delta (B.1.617.2) and epsilon (B.1.427/B.1.429), which have been associated with high transmissibility and disease severity in many geographical regions (58, 59). It is important that NAAT diagnostic tools are routinely quality-checked to ensure that they detect all variants in circulation and meet international regulatory test performance criteria (60).

## Sample Types for the Detection of SARS-CoV-2 RNA

The sensitivity and performance of NAATs for accurate detection of SARS-CoV-2 relies on the specimen type and quality, and the method used for processing the sample (37, 61–65). According to the WHO guidelines, testing for SARS-CoV-2 viral RNA requires respiratory samples. Upper respiratory specimens (nasopharyngeal, nasal, and/or oropharyngeal swabs) are most suited for testing early-stage infections, especially in asymptomatic or mild cases, while lower respiratory specimens (sputum and/or endotracheal aspirate or bronchoalveolar lavage) are recommended if for patients in the post-symptomatic phase of the disease and those with severe disease (2). In addition to respiratory samples, detection of viral RNA in serum and fecal samples collected from infected patients has also been reported, in particular where respiratory specimen gave a negative test result (55, 63, 64). However, these samples provide no clear utility for accurate detection of active SARS-CoV-2 infection (66, 67).

Specimens collected from infected individuals at the pre-symptomatic phase through to the hyperinflammatory phase of COVID-19 have resulted in variable positive rates. Studies have shown that a few days prior to and during the symptomatic phase, sputum and nasopharyngeal swab samples gave higher PCR positivity compared to fecal samples. However, the opposite has been observed during the recovery phase (63, 68), demonstrating the potential utility of fecal samples for monitoring viral clearance during the recovery phase. Although a few studies have been able to recover viable virus from fecal samples and anal swabs of convalescent patients (69–71), it is important to note that the presence of viral RNA in feces may not be an indication of active infection but an indication of residual viral RNA being cleared from the body via shedding of infected epithelial cells.

Recent evidence has demonstrated the utility of sputum and saliva as specimens for detection of SARS-CoV-2 (72–76) (Figure 1). For instance, a comparison of sample positivity using quantitative RT-PCR showed that sputum samples had higher positive rates than throat and nasal swabs collected from the same patient (65). Other studies have also reported differences in test sensitivity comparing saliva and nasopharyngeal swabs (73, 76–81). Saliva has been recommended for COVID-19 diagnosis, in particular for surveillance activities. Saliva sampling is non-invasive and suitable for COVID-19 screening in vulnerable populations and in settings where swabs are in limited supply (79, 82, 83). Sputum offers comparable sensitivity to other respiratory samples for the detection of SARS-CoV-2 RNA (74, 84) but its use is limited in situations where patients are unable to expectorate enough sputum for testing (72, 74). Unless collected properly, sputum sampling poses a high risk of viral transmission. Therefore, nasal swabs are preferred over sputum for the detection of SARS-CoV-2 RNA by NAAT methods (Figure 1).

During the early stages of the pandemic, detection of SARS-CoV-2 infection was severely impacted due to the shortage of RNA extraction kits (85, 86). In certain circumstances, these shortages led to delays in diagnosis, which hampered public health control efforts. To increase accessibility to molecular diagnostic tools for COVID-19, several research laboratories developed and optimized NAAT protocols to simplify and obviate the need for RNA purification (87, 88). More cost-effective molecular tools will be needed for SARS-CoV-2 surveillance during and post-pandemic.

## Point-of-Care NAATs to Control the Spread of SARS-CoV-2

The global spread of SARS-CoV-2 and its associated morbidity and mortality requires cost-effective laboratory equipment and PoC diagnostic tools for screening at-risk populations. PoC tests are easy to use and could be readily deployed at healthcare centers, schools and airports, and among vulnerable populations in aged care centers. RT-PCR is generally performed in centralized Biosafety level 2 (BSL2) laboratories and require regulatory approval to undertake COVID-19 testing (37–39). The complexities and the long wait times (≥2 h) for RT-PCR test results makes it a less attractive tool for PoC diagnosis of COVID-19.

Development of a NAAT assay combining RNA extraction, cDNA synthesis and amplification in a single reaction tube, and without the need for sophisticated instruments offers huge prospects for COVID-19 diagnosis at the point-of-care (89, 90) (Figure 1). iNAATs including RPA and LAMP do not require expensive PCR equipment, tolerate crude lysates as input for amplification and can be integrated into portable isothermal instruments for PoC COVID-19 testing (91) (Figure 1). LAMP and RPA applications for SARS-CoV-2 detection have been reviewed elsewhere (89). Although iNAATs pose a higher risk of cross-contamination when compared to PCR-based diagnostic tools (Table 1), certain strategies have been shown to mitigate this problem (92–94). For instance, a pre-optimized closed-tube isothermal amplification coupled with quality control checks to eliminate false-positive and carry-over contamination would be optimum for SARS-CoV-2 testing (Figure 1). As countries scale-up efforts from control to elimination of SARS-CoV-2, cost-effective molecular tools including iNAAT that require minimal sample processing and can be integrated into portable isothermal devices for use at the point-of-care or in the field will be crucial to elimination efforts.

## Conclusion

Laboratory testing for COVID-19 has been integral to public health efforts to control the spread of SARS-CoV-2 globally. However, the high cost and centralization of RT-PCR testing, and the long testing times from sample collection to receipt of test results could hamper SARS-CoV-2 control efforts. RT-LAMP-based testing methods overcome most of the limitations of RT-PCR and can be developed for PoC diagnosis of COVID-19. Since they are compatible with most sample types for detecting active SARS-CoV-2 infections. Thus, they could complement other low-cost diagnostic tools including RDTs and lateral-flow tests for monitoring SARS-CoV-2 transmission locally and globally. With the emergence of SARS-CoV-2 variants, routine quality control checks of NAAT diagnostic tools will be needed to ensure that they meet regulatory and test performance requirements.

## Author Contributions

CN and JR conceived and designed the review. CN, DS, and DD-D conducted the literature review and wrote majority of the manuscript. LM and JR contributed to literature review and writing. JR and WH critically revised the manuscript. All authors have read and approved the manuscript for publication.

## Funding

This work was supported by the National Health and Medical Research Council (NHMRC) of Australia (APP1161076 to JR). Burnet Institute received funding from the NHMRC Independent Research Institutes Infrastructure Support Scheme, and the Victorian State Government Operational Infrastructure Support Scheme. CN was partly supported by a COVID-19 grant (Ref. No. BSAC-COVID-64) from the British Society for Antimicrobial and Chemotherapy. The funders had no role in study design, data collection and analysis, decision to publish, or preparation of the manuscript.

## Conflict of Interest

ZiP Diagnostics is commercializing a COVID-19 point of care test. CN, DS, DD-D, WH, and JR have employment at ZiP. The remaining author declares that the research was conducted in the absence of any commercial or financial relationships that could be construed as a potential conflict of interest.

## Publisher's Note

All claims expressed in this article are solely those of the authors and do not necessarily represent those of their affiliated organizations, or those of the publisher, the editors and the reviewers. Any product that may be evaluated in this article, or claim that may be made by its manufacturer, is not guaranteed or endorsed by the publisher.
